# EDEN: multiscale expected density of nucleotide encoding for enhanced DNA sequence classification with hybrid deep learning

**DOI:** 10.1186/s12859-026-06367-6

**Published:** 2026-01-24

**Authors:** Saman Zabihi, Sattar Hashemi, Eghbal Mansoori

**Affiliations:** Department of Computer Science, Engineering, and IT, Shiraz University, Shiraz, Iran

**Keywords:** DNA sequence classification, Multiscale sequence encoding, Kernel density estimation, Hybrid convolutional neural network, Unified sequence representation, Genomic deep learning

## Abstract

**Background:**

DNA sequences are fundamental carriers of genetic information, and their accurate classification is essential for understanding gene regulation, disease mechanisms, and translational genomics. Existing encoding methods often fail to capture both local and long-range dependencies simultaneously.

**Results:**

We introduce EDEN (Expected Density of Nucleotide Encoding), a unified multiscale encoding framework based on kernel density estimation. EDEN captures position-specific and context-dependent nucleotide patterns and integrates them into a hybrid deep learning architecture. Across sixteen benchmark datasets covering promoter detection, core promoter detection, and transcription factor binding prediction, EDEN achieves the best average performance while using orders of magnitude fewer parameters compared with state-of-the-art models. All source code, pretrained models, and datasets are publicly available at: https://github.com/zabihis/EDEN.

**Conclusions:**

EDEN provides an efficient, biologically informed, and interpretable multiscale representation for genomic sequence classification. Its favorable parameter-performance ratio and robust consistency across tasks underscore its practicality for large-scale genomic applications.

## Introduction

DNA sequences, as fundamental blueprints of life, govern diverse biological processes. Accurate classification is crucial for understanding biology, disease diagnosis, and drug discovery. While artificial intelligence has revolutionized genomic analysis [1–3], the symbolic and non-numerical nature of these data, along with the substantial amount of rapidly generated biosequence data, presents persistent major challenges for computational methods [4, 5]. Encoding raw nucleotide sequences into meaningful numerical representations therefore remains a critical prerequisite for effective machine learning applications [6].

Current approaches to DNA sequence encoding typically fall into two categories: position-specific methods like one-hot encoding that preserve exact nucleotide locations but lack contextual awareness, and composition-based methods like k-mer frequencies that capture regional patterns but sacrifice spatial precision. Effective sequence analysis requires capturing both local and long-range features. However, many current methods struggle to integrate these features effectively [7], leading to information loss and suboptimal performance [8]. This highlights the need for a position-aware approach capable of integrating patterns from both single-scale and multiscale analyses.

**Fig. 1:**
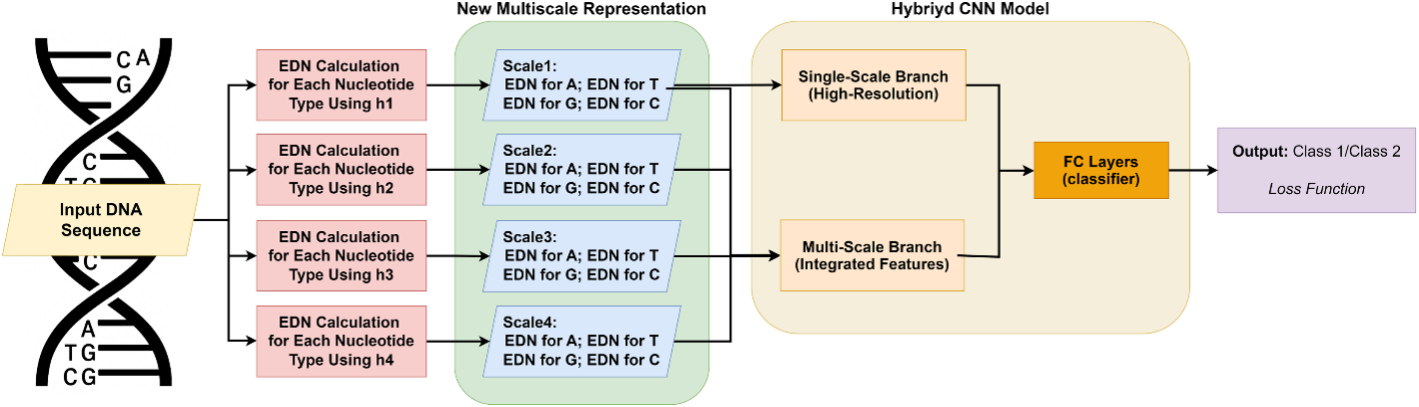
EDEN architecture and encoding pipeline. Generation of multiscale EDN features. For each nucleotide, kernel density estimates with bandwidths *h* = 1,3,5,7 are computed along the sequence, producing position-aware multiscale density maps. These serve as input to the dual-branch hybrid CNN model, which extracts high-resolution and multiscale features for sequence classification

To address this gap, we introduce EDEN, a novel framework that unifies the principles of discrete sequence representations through a numerical, multiscale encoding strategy. At the core of our approach is the EDN representation, which transforms symbolic sequences into spatially-aware density profiles using kernel density estimation (KDE). This representation bridges the conceptual divide between one-hot encoding and k-mer analysis by modeling nucleotide distributions across multiple biologically-relevant scales using a multiscale, spatially-aware encoding strategy based on KDE, effectively capturing both precise positional information and broader compositional contexts within a single, coherent framework.

As illustrated in Fig. 1, the EDEN framework processes DNA sequences through a sophisticated pipeline that generates multiscale EDN features. For each nucleotide type, kernel density estimates with bandwidths corresponding to 1-, 3-, 5-, and 7-mer scales produce position-aware density maps that reflect the local concentration of each nucleotide across the sequence. These rich, multiscale representations are then processed by a specialized hybrid deep convolutional neural network (CNN) featuring dual branches: a high-resolution branch that captures base-level signals and a multiscale branch that integrates broader compositional patterns. By fusing outputs from both branches using two fully-connected layers, our method effectively leverages complementary information for enhanced sequence classification.

We rigorously evaluate the EDEN framework through extensive benchmarking across sixteen datasets encompassing genomic detection and classification tasks, including core promoter detection, promoter detection, and transcription factor binding prediction in human and mouse genomes. Our results demonstrate that EDEN achieves best average performance compared to state-of-the-art methods while utilizing significantly fewer parameters, validating both the efficiency and effectiveness of our encoding approach.

The remainder of this paper is structured as follows: Sect. Related work reviews related work on DNA sequence representation. Section Proposed method: EDEN framework outlines the proposed EDN numerical representation, its multiscale generation, and the hybrid deep learning architecture. Section Experimental setup, results, and discussion presents the experimental setup, results, and discussion. Section Conclusion presents the conclusion and Sect. Future work outlines future research directions.

## Related work

The increasing availability of genomic data has significantly advanced DNA sequence classification. To transform these symbolic sequences into machine-readable formats, various computational methods have been developed. These methods encompass traditional, rule-based or statistical approaches and machine learning and deep learning techniques.

### Traditional sequence representations

The traditional methods for representing biological sequences for computational analysis include the following:

- *Character string encoding* This basic form uses alphabetic characters (e.g., “ACGTATTACGGA”) as raw inputs.
- *Numerical encoding* This assigns unique integers (e.g., A = 0, C = 1, G = 2, T = 3) to nucleotides, providing compact representation but introducing an arbitrary ordinal bias, which lacks biological meaning and can negatively affect model performance by introducing positional bias [9].

- *One-hot encoding* Represents each nucleotide as a 4-dimensional binary vector (e.g., A=[1,0,0,0]). While simple and avoiding ordinal bias, it can be memory-intensive for very long sequences because of sparse binary vectors [9]. As previously said, methods like one-hot encoding preserve exact nucleotide locations but lack contextual awareness.

- *K-mer encoding* This method breaks sequences into fixed-length substrings (k-mers) and uses their frequencies or pattern to extract local patterns and nucleotide combinations [10, 11]. However, increasing *k* leads to high-dimensional feature spaces (“curse of dimensionality”), and k-mer counts often lose long-range dependency information [12–14]. As previously said, composition-based methods like k-mer frequencies capture regional patterns but sacrifice spatial precision.
- *Physicochemical feature encoding* Represents nucleotides by their physical and chemical properties (e.g., hydrogen status, hydrophobicity, and electrical charge) [15]. This numerical approach captures biologically relevant information, though selecting the most relevant features remains a challenge.

### Advanced learning-based representations

Recent advancements, particularly in deep learning and natural language processing, have led to more sophisticated sequence representation methods and larger ML models:

- *Word-based (embedding) representations* Inspired by NLP, these adapt word embedding techniques to represent nucleotides or k-mers. They learn semantic and co-occurrence relationships, mapping elements into a lower-dimensional vector space where similar contexts are closer. While capturing complex relationships, effective training requires very large datasets. Large language models (LLMs) have further revolutionized this area, enabling sophisticated sequence modeling and functional annotation [16, 17].
- *Graph-based representations* Model sequences as graphs where nodes (nucleotides/k-mers) and edges (relationships such as linear adjacency or structural interactions) capture complex connections [18]. The main challenge for tasks such as sequence assembly and multi-omics data analysis [19, 20] is translating linear sequences into appropriate graph structures.
- *Visual representations (image-based)* Convert sequences into 2D images by assigning visual attributes to nucleotides, enabling pattern recognition via image analysis. However, such transformations can introduce information loss or spurious patterns.
- *Fractal representation (e.g., Chaos game representation-CGR)* Maps DNA sequences into fractal patterns where each nucleotide dictates movement in a defined space. While aiding sequence comparison, quantitative feature extraction for ML models can be challenging, often requiring the conversion of CGR coordinates to numerical codes [21, 22].
- *Spectrogram representation* Converts numerical DNA sequences into visual spectrograms to display frequency changes over time [23, 24]. While revealing hidden periodicities, their interpretation requires specialized signal processing knowledge.
- *Latent representations (embedding space)* Learned by neural networks, such as autoencoders (AEs) [25, 26], to encode features into a ‘hidden’ space. AEs learn compact, informative representations by compressing and reconstructing input, with recent work extending this to DNA representation learning [27].
- *Transformer-based models* Inspired by their success in natural language processing (NLP), models based on the transformer architecture have been adapted for DNA sequence analysis. These models, such as DNABERT [28], DNABERT-2 [29], and the Nucleotide Transformer [30], excel at capturing long-range dependencies and global contextual relationships, which is a significant advantage over traditional convolutional or recurrent neural networks. By processing entire sequences or large segments at once, they can learn powerful representations that capture the full context of a DNA sequence, leading to superior performance in various downstream tasks [31].
- *Coding region prediction methods* For coding‑region prediction tasks, methods have been developed to distinguish protein‑coding from non‑coding sequences. Wei et al. [32] introduced a hybrid‑encoding framework that captures global sequence order and gapped k‑mer features, while CPPVec [33] uses distributed representation (doc2vec) of protein sequences to exploit contextual information for coding‑potential prediction.
- *Base-resolution prediction models* Base‑resolution models predict transcription factor binding or chromatin accessibility at single‑nucleotide precision, typically using deep convolutional networks trained on high‑resolution assays (e.g., ChIP‑seq, ATAC‑seq). For instance, BPNet [34] infers base‑resolution ChIP‑nexus profiles and cooperative binding syntax, while FCNsignal [35] employs fully convolutional networks to locate binding sites and motifs genome‑wide. In contrast to these models, which learn motifs and context directly from one‑hot encoded sequences, EDEN provides pre‑computed, multiscale density profiles (EDN) that explicitly encode nucleotide‑concentration landscapes. This makes EDEN well‑suited for sequence‑level classification while offering a pathway toward interpretable, density‑aware base‑resolution modeling.

### Hybrid approaches and the need for explicit multiscale representation

Recent efforts have focused on hybrid approaches, which integrate information from multiple encoding methods for comprehensive representations. For example, combining k-mer frequencies with physicochemical features yields representations that consider both sequence patterns and intrinsic nucleotide properties, enhancing model performance by leveraging complementary information. Recent hybrid deep learning models also combine CNNs, LSTMs, and protein language models for complex biological predictions [36].

Despite these advancements, a significant challenge remains: the crucial absence of explicit multiscale representations that can simultaneously extract and integrate localized, high-resolution details with broader, long-range patterns within a single, coherent framework. While standard one-hot encoding is universal, it treats each position discretely and does not inherently embed multiscale information beyond what a deep model might implicitly learn. Such implicit learning often demands immense computational resources and large datasets and may lack direct biological interpretability. Our proposed method directly addresses this issue by introducing a novel, biologically-informed numerical representation that provides inherent multiscale profiles, thereby enriching deep learning inputs and potentially enhancing efficiency and accuracy in classification tasks while also offering a pathway for future explainable AI (XAI) insights into learned biological patterns [37–39].

## Proposed method: EDEN framework

Our proposed framework introduces a novel approach for DNA sequence encoding and classification. It addresses the challenges of symbolic sequence representation and captures both local and global features by integrating a biologically-informed numerical encoding with a tailored deep learning architecture. This section details our proposed representation, its multiscale generation, and the hybrid deep learning model.

### EDN numerical representation

The EDN method transforms symbolic DNA sequences into a numerical representation. It encodes k-mers by modeling the multiscale density of each nucleotide (A, C, G, T/U) along the sequence’s positional axis via KDE. KDE, a non-parametric method, estimates probability density functions (PDFs) from discrete data, converting them into a smooth, numerical signal vital for capturing spatial distributions and trends. Given a set of *N* data points {x_1_​,x_2_​,…,x_N_​}, the general KDE is expressed as:

1 $$\:\widehat{f}\left(x\right)=\frac{1}{Nh}\sum\:_{i=1}^{N}K\left(\frac{x-{x}_{i}}{h}\right)$$

where f(x) is the PDF, *k* is the kernel function and *h* is the bandwidth, which controls the spatial span of the kernel. The probability that a data point *X* falls within a specified interval [a, b] is given by the integral of its PDF:

2 $$\:P\left(a\le\:X\le\:b\right)=\:\underset{a}{\overset{b}{\int\:}}f\left(x\right)dx$$

For a total of *N* data points, the average number of points expected to fall within a specified region [a, b] is as follows:

$$\:E\left(number\:of\:points\:in\:\left[a,b\right]\right)=N\:\times\:P\left(a\le\:X\le\:b\right)$$

3 $$\:=N\underset{a}{\overset{b}{\int\:}}\:f\left(x\right)\:dx$$

For very small intervals, these relationships can be approximated as follows:

4 $$\:P\left(x\le\:X\le\:x+\:\varDelta\:x\right)\approx\:f\left(x\right)\varDelta\:x$$

5 $$\:E\left(Equation\,Number\:of\:points\:in\:\left[x,\:\:x+\varDelta\:x\right]\right)\approx\:N.f\left(x\right).\varDelta\:x$$

**Fig. 2:**
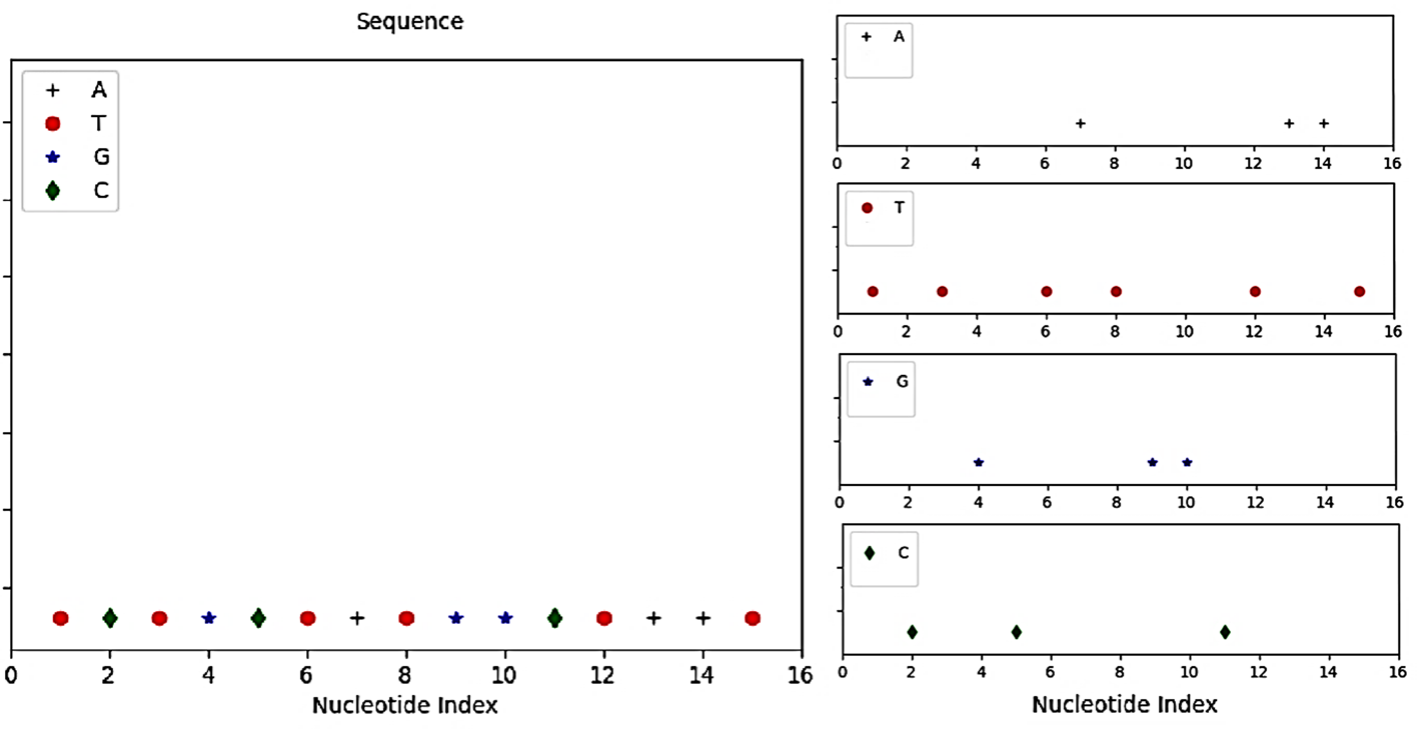
Left: Sample sequence TCTGCTATGGCTAAT; Right: Four distinct nucleotide spaces in a Cartesian coordinate system

Dividing Eq. (5) by *Δx* yields a fundamental relationship that represents the expected density of points in the immediate vicinity of *x* and by considering the nucleotides as the data points:

6 $$\:\mathrm{E}\mathrm{D}\mathrm{N}\left(x\right)=\:E\left(density\:of\:points/nucleotide\:around\:x\right)=\mathrm{N}\:.\:f\left(x\right)$$

The EDN should be calculated for each nucleotide type separately. For this purpose, the spatial regions corresponding to A, C, G, and T are separated as shown in Fig. 2 and considered independently. This segregation is crucial for accurately modeling the density of each nucleotide independently. Each nucleotide retains its original positional index in the sequence along the axis. We calculate the EDN for each nucleotide type within its distinct spatial space, yielding EDN_A_, EDN_T_, EDN_G,_ and EDN_C_. This process effectively converts discrete nucleotide positions into a smooth, density-based signal that inherently captures localized spatial context. Leveraging the concept from Eq. (6), the EDN for each specific nucleotide type Z ∈ {A, C, G, T} at position *x* is defined as:

7 $$\:{EDN}_{A}\left(x\right)={N}_{A}.{f}_{A}\left(x\right)=\frac{1}{h}\sum\:_{i=1}^{{N}_{A}}K\left(\frac{x-{x}_{i}}{h}\right)$$

8 $$\:{EDN}_{T}\left(x\right)={N}_{T}.{f}_{T}\left(x\right)=\frac{1}{h}\sum\:_{i=1}^{{N}_{T}}K\left(\frac{x-{x}_{i}}{h}\right)$$

9 $$\:{EDN}_{G}\left(x\right)={N}_{G}.{f}_{G}\left(x\right)=\frac{1}{h}\sum\:_{i=1}^{{N}_{G}}K\left(\frac{x-{x}_{i}}{h}\right)$$

10 $$\:{EDN}_{C}\left(x\right)={N}_{C}.{f}_{C}\left(x\right)=\frac{1}{h}\sum\:_{i=1}^{{N}_{C}}K\left(\frac{x-{x}_{i}}{h}\right)$$

Here, EDN_z_​(x) represents the EDN for nucleotide Z at position *x*, N_z_​ is the total count of nucleotide Z within the sequence, f_z_​(x) is its estimated PDF and *x*_*i*_ is the spatial index of the i-th nucleotide Z.

As previously defined, EDN signals are inherently numerical and continuous. For training a machine learning model using the proposed representation, in this study, the EDN values are computed only at the position of each nucleotide along the input sequence. The EDN values at these discrete points constitute the numerical encoding of the sequence at those respective positions. To this end, we use Eq. (11) to calculate the new representation:

11 $$\:R=\:{EDN}_{A}\left(x\right)\:\left|\right|\:{EDN}_{T}\left(x\right)\:\left|\right|\:{EDN}_{G}\left(x\right)\:\left|\right|\:{EDN}_{C}\left(x\right)\:\:\:for\:x\: \epsilon \:[1,\:2,\:\dots\:,N]$$

Here, *R* is the proposed representation, *x* is the positional coordinate along the axis, *N* is the length of the sequence and $\:[1,\:2,\:\dots\:,N]$ represents the positional indices of the nucleotides in the sequence.

The EDN signal can be understood intuitively as a measure of local nucleotide concentration along the sequence. For example, a high EDN_A_(x) value at position *x* indicates that Adenine is relatively abundant in the genomic neighborhood around *x*, as defined by the kernel bandwidth *h*. Smaller values of *h* (e.g., *h* ~ 1-mer) reflect the immediate presence or absence of A at precise positions, akin to a smoothed one-hot signal. Larger *h* values (e.g., *h* ~ 5-mer) capture the broader prevalence of A over a region, analogous to measuring the A-richness of a sequence window—without losing positional awareness. This representation therefore bridges discrete nucleotide identity and regional composition, providing a continuous, spatially-aware profile of each nucleotide type’s distribution.

### Multiscale EDN generation using K-mer concept

The EDEN framework introduces a unified approach to DNA sequence representation by generalizing and integrating the fundamental principles of one-hot and k-mer concept using a multiscale, spatially-aware encoding strategy based on KDE. This is achieved through the KDE parameter *h* (bandwidth), which controls the spatial context for nucleotide density calculation.

#### A unified framework for sequence encoding

The EDN representation creates a continuum between traditional encoding methods through its bandwidth parameter:

- At minimal bandwidth ($\:\boldsymbol{h}\sim\:1$), EDN approximates a smoothed one-hot encoding, capturing the precise identity of nucleotides at specific positions while maintaining spatial continuity. This provides high-resolution positional information analogous to traditional one-hot encoding, but in a numerical space that avoids discrete binary representations.
- At increased bandwidths ($\:\boldsymbol{h}>1$), EDN generalizes to embody the principles of k-mer analysis, capturing nucleotide composition over broader neighborhoods equivalent to 3-mer, 5-mer, and 7-mer contexts. However, unlike conventional k-mer counting that loses positional information, EDN preserves spatial relationships while modeling regional nucleotide densities.

EDN is a unified approach that overcomes key limitations of both methods: it maintains the positional precision of one-hot encoding while incorporating the contextual awareness of k-mer analysis, all within a multiscale strategy based on KDE suitable for deep learning.

#### Biologically-informed multiscale representation

The selection of specific bandwidths corresponding to k-mer lengths (1, 3, 5, and 7) is biologically motivated, capturing genomic features at their characteristic scales:

- $\:\boldsymbol{h}\sim\:1$**-mer**: Captures single-nucleotide polymorphisms and position-specific nucleotide preferences.
- $\:\boldsymbol{h}\sim\:3$**-mer**: Models codon-level patterns and core transcription factor binding motifs.
- $\:\boldsymbol{h}\sim\:5$**-mer**: Represents typical transcription factor binding site lengths and composite regulatory elements.
- $\:\boldsymbol{h}\sim\:7$**-mer**: Captures broader domain-level features including nucleosome positioning signals.

By concatenating representations across these four scales, EDEN provides the neural network with a comprehensive feature set spanning the full spectrum of biological organization—from individual nucleotide identity to regional sequence composition.

The selection of odd values for k-mer, ensures symmetric neighborhood consideration around each position, maintaining positional integrity while providing balanced contextual information. This systematic scaling enables the simultaneous extraction of features ranging from highly localized nucleotide arrangements to diffuse, long-range compositional patterns.

#### KDE analysis and bandwidth selection

In KDE, the selection of both the kernel function and bandwidth parameter critically influences the resulting density representation. The kernels employed in this study—cosine, linear, exponential, and Gaussian—share the fundamental property of symmetry around their center, with magnitude maximized at the central position and smoothly decaying with increasing distance.

For bandwidth selection, we implemented a principled criterion where the bandwidth parameter *h* was specifically calibrated such that each kernel’s magnitude decays to exactly 50% of its maximum value at the boundary positions of the k‑mer window. This 50% decay threshold—which corresponds to choosing the 0.5 point as the effective cutoff for a soft window that is 1 at the center and 0 at the tails—provides a natural, consistent, and interpretable criterion for defining the effective neighborhood. It represents the point where the kernel’s influence is equally balanced between the central position and the window boundary, ensuring a symmetric and interpretable transition of influence across the defined k‑mer scale. We applied the same logic to determine the effective boundary of each kernel in the density estimation, thereby defining the number of nucleotides that fall within the kernel’s influential range. This defines the effective domain of influence for each kernel, ensuring consistent and biologically meaningful neighborhood definitions across different k‑mer scales.

This principled criterion ensures that each kernel’s influence is appropriately scaled to the biological feature of interest (e.g., 3‑mer for motifs, 7‑mer for nucleosome signals), providing a fixed, interpretable, and reproducible multiscale representation without requiring dataset‑specific tuning. Although the chosen threshold is simple, the model delivers the desired performance; in line with Occam’s razor, the straightforward solution that yields acceptable results is preferred over more complex alternatives. While adaptive bandwidth learning represents an interesting future direction, the current heuristic is sufficient and effective for capturing the range of regulatory scales examined in this study.

**Fig. 3:**
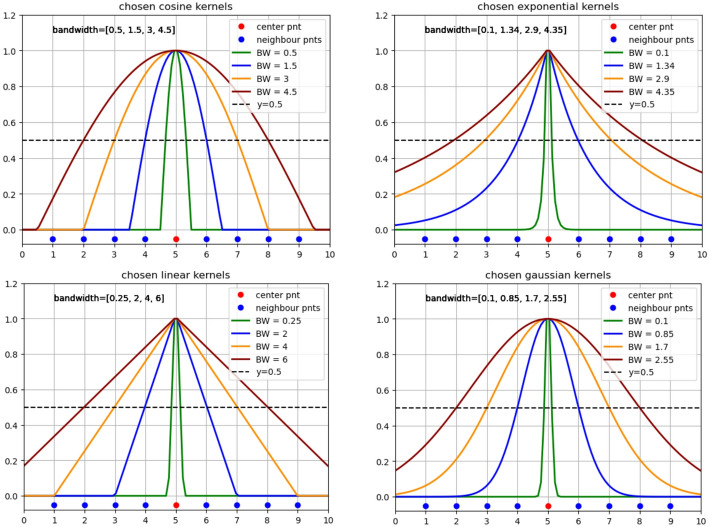
Kernel bandwidth calibration for multiscale EDN encoding. The bandwidth parameter *h* for each kernel (Cosine, Linear, Exponential, Gaussian) was systematically determined by setting its value such that the kernel’s magnitude decays to 50% of its maximum value (dashed horizontal line) at the boundaries of the k-mer window. This consistent criterion ensures that each kernel’s effective domain of influence corresponds precisely to the biological scale of the target k-mer length (1, 3, 5, and 7), enabling the generation of comparable and biologically meaningful multiscale density representations

As illustrated in Fig. 3, this criterion was systematically applied to determine the appropriate bandwidth values for each kernel type (cosine, linear, exponential, and Gaussian) across the four k-mer scales (1, 3, 5, and 7). The horizontal reference line at y = 0.5 confirms that all kernels consistently achieve the specified 50% decay threshold at their respective window boundaries, validating the uniform application of our bandwidth selection criterion.

This systematic approach to bandwidth calibration ensures that the resulting EDN representations maintain consistent spatial characteristics across different kernel types while capturing nucleotide density patterns at biologically relevant scales.

#### Fundamental Distinction from Convolutional Operations

While EDN and convolutional operations both process local sequence neighborhoods, they represent fundamentally different approaches:

- EDN as unified representation learning:
  - Provides an interpretable, numerical, biologically-informed multiscale representation that generalizes one-hot encoding and k-mer pattern principles using encoding strategy based on KDE.
  - Captures general nucleotide density landscapes across multiple biological scales.
  - Serves as an explicit feature engineering step that structures input for subsequent pattern recognition.
- Convolution as adaptive pattern detection:
  - Learns task-specific motif detectors through training.
  - Discovers discriminative patterns from unstructured inputs that necessarily are not interpretable and biologically-informed.
  - Must implicitly discover relevant scales from raw data.

This distinction is crucial: The EDN encoding is a biologically intuitive, feature-engineered representation that transforms raw sequences into structured multiscale landscapes that explicitly represent the continuum from nucleotide-specific to context-compositional information. This preprocessing allows the subsequent convolutional network to focus on detecting higher-order interactions within this enriched, biologically-structured feature space, rather than learning basic sequence properties from scratch.

#### Computational efficiency and representation properties

**Fig. 4:**
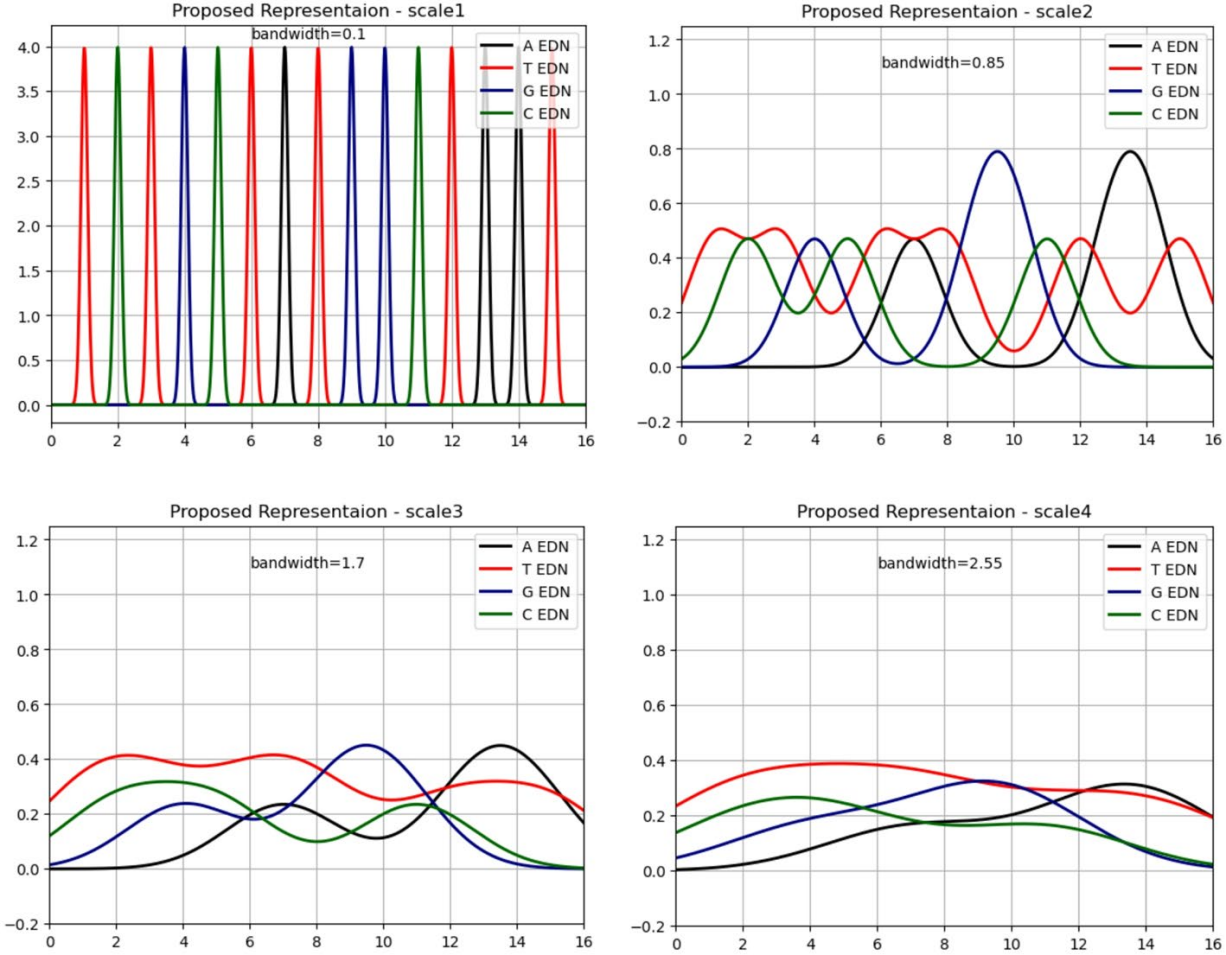
EDN signal visualization for sequence TCTGCTATGGCTAAT. Position-wise density profiles for each nucleotide type across four bandwidth scales (1-, 3-, 5-, 7-mer), demonstrating how EDN captures localized nucleotide concentrations and broader compositional patterns

The concatenation of EDN representations across scales (Scale 1 to Scale 4) produces a comprehensive feature set that maintains several advantageous mathematical properties. As demonstrated in Fig. 4, which shows the EDN representation for sequence “TCTGCTATGGCTAAT” across four bandwidths, the expected value for the sum of the four nucleotide density signals at any position equals one:

$$\:{EDN}_{A}\left(x\right)+{EDN}_{T}\left(x\right)+{EDN}_{G}\left(x\right)+{EDN}_{C}\left(x\right)=1$$

This theoretical normalization property ensures stable numerical behavior and interpretable feature magnitudes across different sequence contexts.

The computational complexity of multiscale EDN generation remains linear with sequence length (O(N)), making it scalable for genome-wide applications. By employing odd *k* for k-mer and strategic bandwidth selection, we achieve comprehensive multiscale coverage while minimizing redundant computations, ensuring both biological relevance and computational efficiency.

These multiscale EDN representations serve as powerful feature engineering tools that explicitly encode biologically meaningful sequence properties, reducing the representational burden on the downstream neural network and enabling more efficient learning of complex genomic patterns.

### Hybrid deep learning architecture

To effectively leverage the proposed multiscale representations, we propose a novel hybrid deep CNN architecture. This design ensures comprehensive pattern recognition by integrating both high-resolution, single-scale features and broader multiscale information. As shown in Fig. 1, the architecture comprises two distinct branches, each tailored to process various aspects of the EDN-encoded data:

- *High-resolution branch (HFBranch)* This single-scale branch processes Scale 1 of the EDN representations, which corresponds to a 1-mer. It focuses on extracting localized, high-resolution features from nucleotide density profiles, capturing intricate details of 1-mers and short-range dependencies.
- *Multiscale branch (integrated features)* This branch processes a concatenation of EDN representations generated with multiple kernel bandwidths (*h*). Each bandwidth corresponds to a distinct biological scale (1-mer, 3-mer, 5-mer, or 7-mer), allowing this branch to simultaneously capture a spectrum of density patterns ranging from local to more global. This integrated input enables the network to learn robust features that combine information from various scales within a single processing pathway.

The outputs from both the single-scale and multiscale branches are then fused via a fully-connected layer. This fusion layer is crucial for leveraging the complementary information learned by each branch. By combining detailed local patterns with broader multiscale contextual features, our hybrid architecture enhances the model’s ability to identify complex biological signals and achieves superior classification performance in DNA sequence analysis.

**Table 1:** Detailed architecture of the EDEN hybrid CNN model. Layer specifications, parameter counts, and design rationale for the high-resolution and multiscale branches and classifier

Branch	Layer Type	Output Channels	Kernel Size	Params
High-Resolution	Conv1d	64	5	1.3k
	Conv1d	128	5	41k
	Conv1d	128	5	82k
Multiscale	Conv2d	64	(3,3)	2.4k
	Conv2d	128	(5,5)	205k
	Conv1d	128	5	82k
Fusion & Classifier	Linear	256	-	656k
	Linear	1	-	0.3k

#### Architectural specifications and parameter efficiency

To provide transparency regarding the model’s design and parameter efficiency, Table 1 details the complete EDEN hybrid CNN architecture. The high-resolution branch comprises three 1D convolutional layers (64, 128, and 128 filters) with kernel size 5, ReLU activations, max pooling, and dropout (*p* = 0.2). The multiscale branch employs an initial 2D convolutional layer (64 filters, kernel size 3 × 3) to jointly process spatial and scale dimensions, followed by a second 2D convolution (128 filters, kernel size 5 × 5) and a final 1D convolutional layer (128 filters, kernel size 5). Both branches are adaptively pooled to a fixed length of 10, concatenated, and processed through two fully-connected layers (256 and 1 units) for classification. This design yields a total of 1.07 M trainable parameters.

The parameter efficiency stems from deliberate architectural choices: moderate filter counts (64–128 channels), small kernel sizes (3–5), efficient multiscale processing via 2D convolutions, and a simple fusion strategy using concatenation followed by a single dense layer. These design decisions contrast with deeper genomic CNNs that often employ 256–512 + filters, larger kernels, or complex multi-branch fusion modules.

### Biological interpretation of EDN

EDN representation transforms nominal DNA sequences into numerical spatial density profiles that carry direct biological significance. This transformation can be understood through several complementary biological interpretations:

#### Nucleotide concentration landscapes

At its core, EDN models the local concentration landscape of each nucleotide type along the sequence. For any position *x*, the EDN_A_(x) value represents the relative prevalence of Adenine in the genomic neighborhood defined by the kernel bandwidth *h*. Unlike raw k-mer counts that lose precise positional information, EDN preserves position while summarising neighborhood composition. This is analogous to measuring the “functional density” of biological elements:

- High EDN_G_ peaks often correspond to GC-rich regions, which are characteristic of gene promoters, CpG islands, and stable genomic regions.
- Extended EDN_A_ plateaus may indicate poly-A signals or AT-rich regulatory regions.
- Coordinated EDN_C_/EDN_G_ oscillations can reveal nucleosome positioning patterns or chromatin accessibility signals.

#### Multiscale biological feature detection

The multiscale nature of EDN directly mirrors the hierarchical organization of genomic regulatory elements:

- Fine-scale resolution (*h* ~ 1-mer) captures individual nucleotide preferences and single-base transcription factor binding specificities.
- Intermediate scales (*h* ~ 3–5 mer) correspond to core transcription factor binding motifs (e.g., TATA box, E-box) and short regulatory sequences.
- Broader scales (*h* ~ 7 + mer) capture composite regulatory domains, nucleosome positioning patterns, and regional sequence composition biases.

This multiscale approach enables the detection of both precisely localized motifs and diffuse regulatory domains within a unified framework.

#### Spatial awareness and fuzzy motif representation

Unlike discrete k-mer approaches that require exact positional matching, EDN representation accommodates biological variation in functional element positioning:

- Fuzzy binding sites with variable spacing between half-sites.
- Degenerate motifs where the exact nucleotide sequence varies but the local composition remains conserved.
- Positional tolerance in regulatory elements where the exact position may shift while maintaining functional capacity.

This spatial flexibility mirrors the biological reality that many regulatory elements exhibit positional and sequence degeneracy while maintaining functional conservation.

#### Relationship to known biological patterns

The EDN representation naturally captures several well-established genomic patterns:

- *Nucleosome positioning* The ~ 147 bp periodicity of AA/TT/AT dinucleotides creates characteristic oscillatory patterns in EDN signals.
- *Promoter features* TATA boxes manifest as sharp EDN_A_/EDN_T_ peaks, while CpG islands appear as broad EDN_C_/EDN_G_ plateaus.
- *Transcription factor binding* Different TF families create distinct EDN signatures based on their binding preferences (e.g., zinc fingers vs. helix-turn-helix proteins).

#### Functional interpretation of EDN signals

The EDN signals allow for intuitive biological interpretation:

- Signal amplitude reflects the strength or conservation of a biological feature.
- Signal width indicates the spatial extent of a regulatory element.
- Phase relationships between nucleotide channels reveal coordinated compositional patterns.

This biological interpretability provides a foundation for explainable AI in genomic analysis, as EDN features can be directly mapped to known biological phenomena rather than remaining as abstract numerical representations.

The EDN framework thus bridges the gap between raw sequence data and biological function by providing a spatially-aware, multiscale representation that naturally aligns with the organizational principles of genomic regulatory systems.

### Complexity analysis

The proposed EDN method is designed to be highly efficient and scalable, a critical factor for processing the vast volumes of genomic data being generated. The time complexity of encoding a DNA sequence of length *N* is O(N). This is because the process involves a single pass through the sequence, where each nucleotide is assigned a numerical value through a constant number of operations. Similarly, the space complexity is also O(N), as the output is a numerical vector with a size directly proportional to the input sequence length. This linear scaling ensures that the method can be applied to very long genomic sequences without incurring a significant computational or memory bottleneck, making it a robust and practical solution for large-scale bioinformatics applications.

## Experimental setup, results, and discussion

This section details the experimental design, implementation, and evaluation of the proposed EDEN framework for DNA sequence classification. We compared our model’s performance against several state-of-the-art methods, including the DNABERT-2 model [29] (117 M parameters), four variants of the NT model [30] (with 500 M and 2500 M parameters), four variants of the DNABERT model [28] (86 M parameters) and two fundamental baseline methods: (i) OneHot + HFBranch (standard one-hot encoding processed by an identical CNN architecture) and (ii) K-merFreq + XGBoost (traditional 1-mer, 2-mer, and 3-mer frequency features with gradient-boosted trees). This comprehensive evaluation was conducted on several benchmark datasets.

**Table 2:** Details of benchmark datasets from the Genome Understanding Evaluation (GUE) [29] benchmark used for evaluating EDEN framework performance

Species	Task	Datasets	$\:{\boldsymbol{N}}_{\boldsymbol{C}\boldsymbol{l}\boldsymbol{a}\boldsymbol{s}\boldsymbol{s}\boldsymbol{e}\boldsymbol{s}}$	Seq Len	$\:{\boldsymbol{N}}_{\boldsymbol{t}\boldsymbol{o}\boldsymbol{t}\boldsymbol{a}\boldsymbol{l}}$	$\:{\boldsymbol{N}}_{\boldsymbol{t}\boldsymbol{r}\boldsymbol{a}\boldsymbol{i}\boldsymbol{n}}$
Human	Core Promoter Detection (H-CPD)	PROM_CORE_ALL	2	70	59.2k	47.4k
		PROM_CORE_TATA			6.13k	4.9k
		PROM_CORE_NOTATA			53.1k	42.5k
	Promoter Detection (H-PD)	PROM_300_ALL	2	300	59.2k	47.4k
		PROM_300_TATA			6.13k	4.9k
		300_NOTATA			53.1k	42.5k
	Transcription Factor Prediction (H-TFP)	HUMAN_TF0	2	100	34.4k	32.4k
		HUMAN_TF1			32.7k	30.7k
		HUMAN_TF2			21k	19k
		HUMAN_TF3			29.3k	27.3k
		HUMAN_TF4			21k	19k
Mouse	Transcription Factor Prediction (M-TFP)	MOUSE_TF0	2	100	8.1k	6.48k
		MOUSE_TF1			67.4k	54k
		MOUSE_TF2			3.28k	2.62k
		MOUSE_TF3			2.38k	1.9k
		MOUSE_TF4			18.8k	15.1k

Datasets include core promoter detection (H-CPD), promoter detection (H-PD), and transcription factor binding prediction (H-TFP, M-TFP) tasks in human and mouse genomes

### Datasets

For a robust and comprehensive evaluation, we employed sixteen distinct datasets from the Genome Understanding Evaluation (GUE) benchmark introduced in [29]. The datasets of this benchmark derived from human and mouse cells encompass four DNA classification tasks. The details of these datasets are presented in Table 2. Each dataset includes predefined training, validation, and test subsets to ensure fair comparisons. The primary evaluation metric of this benchmark is the Matthews correlation coefficient (MCC).

### Experimental setup

Here, we detail the implementation of the proposed EDEN framework, including the software and hardware used, as well as the specific parameters for the training process. The entire framework, including the EDN encoding and the hybrid deep learning architecture, was implemented in Python 3 via the PyTorch framework. The models were trained via the binary cross-entropy loss function. To prevent overfitting, an early stopping mechanism was implemented based on the MCC with a patience of four epochs. All the computational experiments were conducted on a machine equipped with a single NVIDIA T4 GPU, 13 GB of system RAM, and 112 GB of disk space. For smaller datasets, the evaluation could be performed efficiently without a GPU.

### Kernel selection

To assess the impact of different kernels on the quality of encoding, the generated numerical features, and the performance of the classifier model, we conducted an evaluation in this section using the H-CPD_CORE_TATA dataset. The results of this evaluation are reported in Table 3. As is evident from these results, the cosine kernel achieved the highest performance on the given dataset, indicating the superior quality of encoding and features derived from this kernel. Therefore, we utilize the cosine kernel in all subsequent analyses.

**Table 3:** Performance comparison of EDEN using different kernel functions for EDN encoding on the H-CPD_CORE_TATA dataset

EDN Kernel	Model Accuracy
cosine	0.9054
exponential	0.8989
gaussian	0.8956
linear	0.8940

Accuracy values demonstrate the impact of kernel selection on classification performance

### Ablation study

To analyze the proposed architecture and investigate the impact of each model branch (high-resolution single-scale analysis and multiscale analysis), we evaluate the model’s performance in three setups via ablation analysis:

- *Setup 1* The model includes only the high-resolution branch, processing single-scale (1-mer) EDN features.
- *Setup 2* The model includes only the multiscale branch, processing concatenated EDN features across all four scales (1-, 3-, 5-, 7-mer).
- *Setup 3* The complete EDEN architecture incorporates both the high-resolution and multiscale branches.

In this section, the evaluation is conducted on the H-CPD_CORE_TATA dataset. As shown in Table 4, the model with Setup 3, which simultaneously incorporates both multiscale and high-resolution single-scale analysis, achieved the highest performance. Furthermore, the model with only multiscale analysis (Setup 2) demonstrated better performance than the model with only single-scale analysis (Setup 1). This evaluation highlights the importance of incorporating high-resolution single-scale analysis alongside multiscale analysis and confirms our proposed model architecture.

**Table 4:** Ablation study results evaluating the contribution of different architectural components in EDEN

Model Configuration	High Resolution	Multiscale	Params	Model Accuracy
Setup1	✓	×	452 K	0.8740
Setup2	×	✓	617 K	0.8889
Setup3	✓	✓	1.069 M	0.9054

Setup 1: high-resolution branch only; setup 2: multiscale branch only; setup 3: both branches (proposed configuration)

### Comparison with state-of-the-art methods

To provide a comprehensive and synthesized overview of model performance, we present a consolidated analysis of results across all sixteen datasets and four genomic tasks. To directly isolate the contribution of the proposed EDN encoding and ensure a fair architectural comparison, we introduce two fundamental baseline models: OneHot + High-Frequency Branch (OneHot + HFBranch), which employs standard one-hot encoding processed by an identical CNN model (the high-resolution branch of EDEN), and K-merFreq + XGBoost, which uses a traditional 1-mer, 2-mer, and 3-mer frequency vector with a gradient-boosted tree classifier (max depth = 6, random state = 42). These baselines serve two purposes: (1) isolating the specific contribution of the EDN encoding from the CNN architecture, and (2) providing direct comparisons with two standard genomic encoding methods: one-hot encoding and k-mer frequencies. Table 5 summarizes the key performance metrics, including mean Matthews Correlation Coefficient (MCC) values for all evaluated methods. For models where we performed multiple runs (EDEN and the OneHot + HFBranch baseline), results are shown as mean ± standard deviation from five independent evaluations. For the K‑merFreq + XGBoost baseline, results are reported without standard deviation as the method is fully deterministic—with fixed hyperparameters and consistent training/validation splits, it produced identical performance across all five independent runs. For state-of-the-art models, we report the MCC values as provided in their respective studies.

**Table 5:** Performance comparison of EDEN and state-of-the-art methods across genomic classification tasks

Method	H-CPD (MCC)	H-PD (MCC)	H-TFP (MCC)	M-TFP (MCC)
EDEN (ours)	**73.65 ± 0.39**	86.53 ± 0.73	63.39 ± 0.76	66.20 ± 0.71
OneHot + HFBranch	71.53 ± 0.15	83.45 ± 0.23	60.84 ± 0.12	60.16 ± 0.88
K-merFreq + XGBoost	66.10	57.86	57.08	39.64
DNABERT-2	70.52	84.21	**70.10**	**67.99**
NT-500 M-human	66.54	85.51	50.82	45.24
NT-500 M-1000 g	69.13	86.58	58.92	49.31
NT-2500 M-1000 g	68.17	86.61	61.99	56.82
NT-2500 M-multi	71.62	**88.14**	63.32	67.01
DNABERT (3-mer)	72.96	84.63	64.43	57.73
DNABERT (4-mer)	71.10	82.99	64.40	59.58
DNABERT (5-mer)	72.03	84.04	50.46	54.85
DNABERT (6-mer)	71.81	81.70	64.17	56.43

Matthews correlation coefficient (MCC) values for core promoter detection (H-CPD), promoter detection (H-PD), human transcription factor prediction (H-TFP) and mouse transcription factor prediction (M-TFP). Values for EDEN and the OneHot + HFBranch baseline are reported as mean ± standard deviation from five independent evaluation runs; other values are as reported in the respective studies. EDEN consistently outperforms the fundamental baselines and shows competitive performance with much larger models

**Fig. 5:**
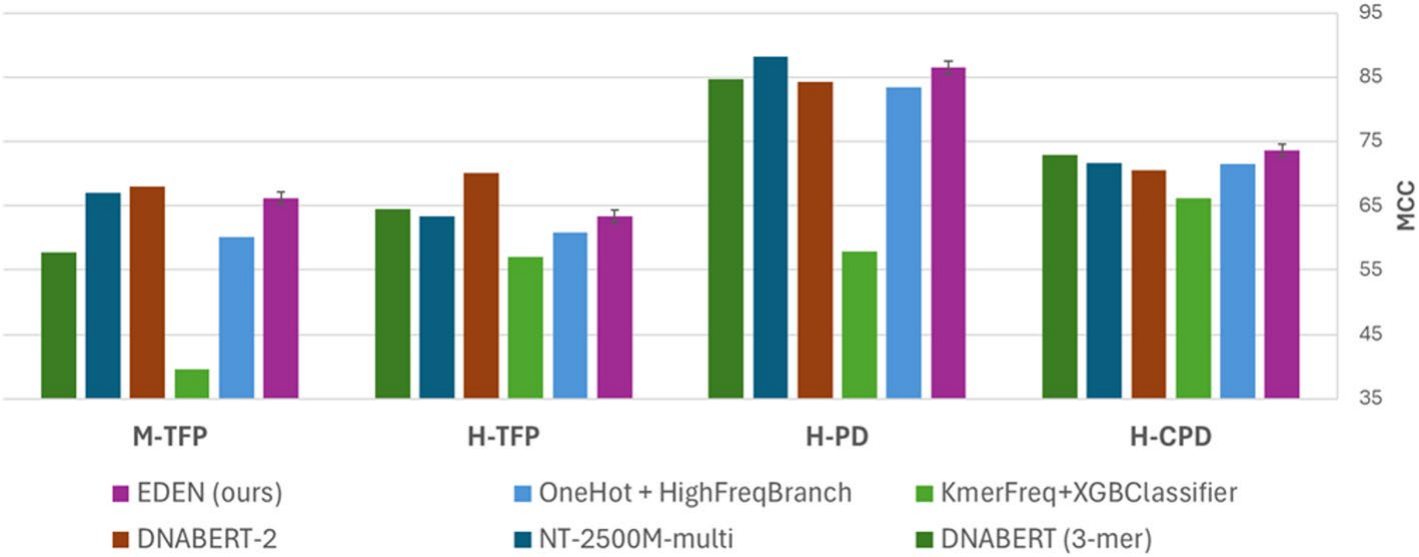
MCC comparison of EDEN against key baselines and state-of-the-art models. Grouped bar chart showing the mean MCC from five independent evaluation runs for a focused set of models: EDEN, the fundamental baselines (OneHot + High-Frequency Branch, K-merFreq + XGBoost), and the best-performing state-of-the-art models (DNABERT-2, NT-2500 M-multi, DNABERT (3-mer)). EDEN consistently outperforms the baselines and achieves competitive results with models that are orders of magnitude larger

The consolidated results clearly demonstrate EDEN’s robust and reproducible performance across diverse classification challenges. As shown in Table 5 and visualized in the focused comparison of Fig. 5, EDEN demonstrates robust performance across all four classification tasks: core promoter detection (H‑CPD, MCC: 73.65 ± 0.39), promoter detection (H‑PD, MCC: 86.53 ± 0.73), human transcription factor prediction (H‑TFP, MCC: 63.39 ± 0.76), and mouse transcription factor prediction (M‑TFP, MCC: 66.20 ± 0.71). The relatively small standard deviations across the five independent runs confirm the statistical stability of EDEN’s performance. EDEN consistently and significantly outperforms both fundamental baselines across all tasks, with particularly notable advantages in promoter-related tasks.

**Table 6:** Performance ranks and parameter efficiency derived from five independent evaluation runs

Method	Rank on H-CPD	Rank on H-PD	Rank on H-TFP	Rank on M-TFP	Average Rank	Params
EDEN (ours)	1	4	5	3	3.25	1.069 M
OneHot + HFBranch	6	9	8	4	6.75	452 K
K-merFreq + XGBoost	12	12	10	12	11.5	N/A
DNABERT-2	8	7	1	1	4.25	117 M
NT-500 M-human	11	5	11	11	9.5	500 M
NT-500 M-1000 g	9	3	9	10	7.75	500 M
NT-2500 M-1000 g	10	2	7	7	6.5	2500 M
NT-2500 M-multi	5	1	6	2	3.5	2500 M
DNABERT (3-mer)	2	6	2	6	4	86 M
DNABERT (4-mer)	7	10	3	5	6.25	86 M
DNABERT (5-mer)	3	8	12	9	8	86 M
DNABERT (6-mer)	4	11	4	8	6.75	86 M

Ranks (1 = best) are calculated from the mean MCC values reported in Table 5 for core promoter detection (H‑CPD), promoter detection (H‑PD), human transcription factor prediction (H‑TFP) and mouse transcription factor prediction (M‑TFP). The inclusion of the fundamental baselines (OneHot + High-Frequency Branch, K‑merFreq + XGBoost) highlights the specific contribution of the EDN encoding. EDEN achieves a strong average rank (3.25) while using orders of magnitude fewer parameters than state-of-the-art models

**Fig. 6:**
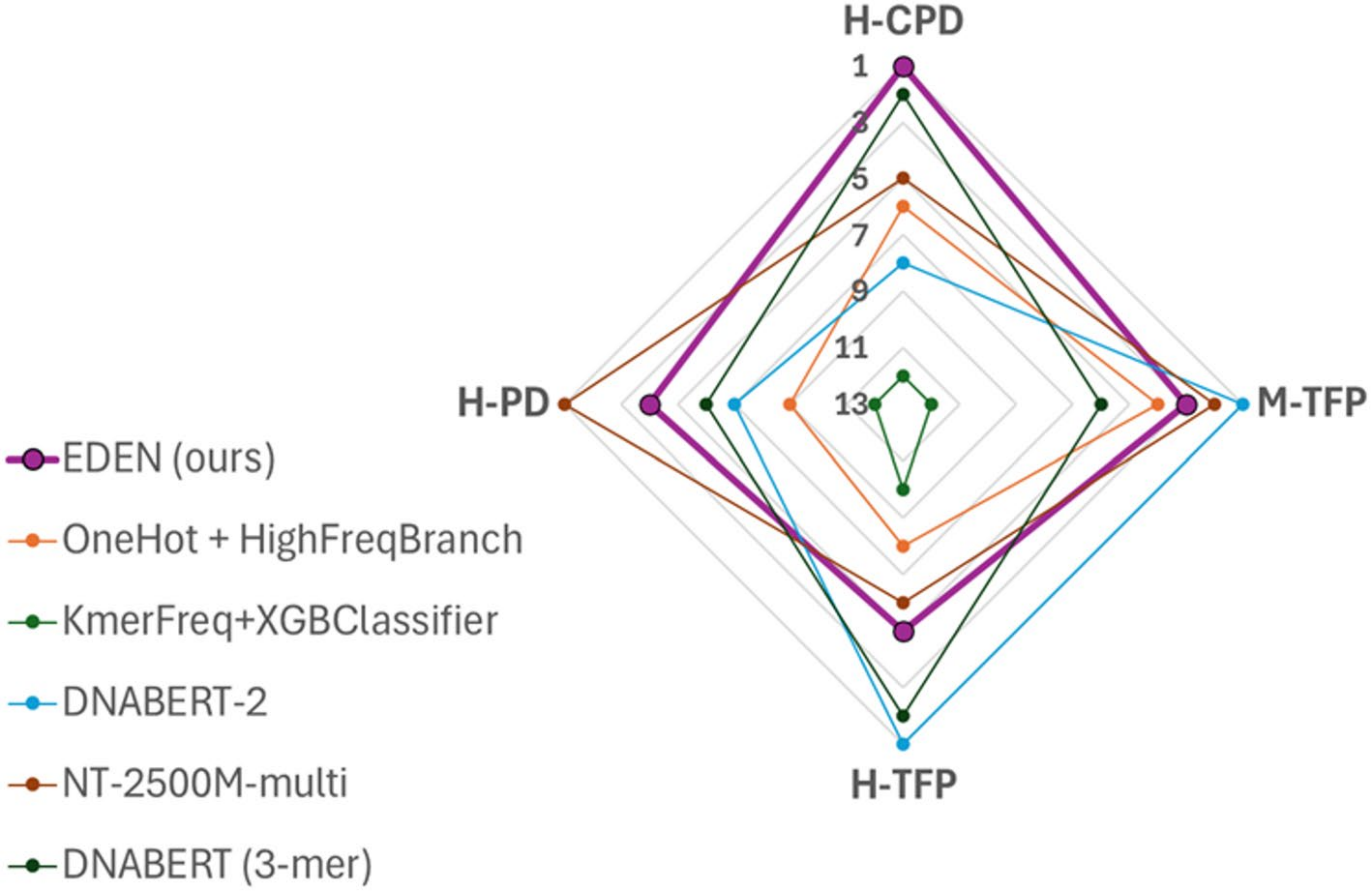
Radar chart of performance ranks based on mean MCC values. The chart visualizes the performance ranks (1 = best) derived from the mean MCC for the same six models shown in Fig. 5. Superior and balanced performance is visualized as a larger and more symmetric polygon in the radar chart

A key finding, evident in the rank analysis of Table 6 and its visual summary in Fig. 6, is that EDEN substantially outperforms both fundamental baselines while competing closely with the best overall models. EDEN attains a strong average rank of 3.25, which is superior to the OneHot + HFBranch baseline (average rank: 6.75) and far exceeds the K-merFreq + XGBoost baseline (average rank: 11.5). Notably, EDEN’s average rank is only marginally behind the top-ranked model, NT-2500 M-multi (average rank: 3.5). This performance gap demonstrates that the gains are not attributable solely to the convolutional architecture or to a standard k-mer featurization, but are intrinsically linked to the novel EDN encoding, which provides a richer, spatially-aware input representation.

EDEN achieves its strong average rank while utilizing approximately 110× fewer parameters than DNABERT-2 (117 M), 470× fewer than NT-500 M variants, and over 2,300× fewer than the largest Nucleotide Transformer model (2500 M). This favorable parameter-to-performance ratio stems from both architectural efficiency and the EDN encoding. As detailed in Table 1, EDEN’s hybrid CNN employs moderate filter counts (64–128 channels), small kernels (size 3–5), and simple fusion, avoiding the parameter inflation common in deeper genomic CNNs. The OneHot + HFBranch baseline (452 K parameters) confirms that architectural compactness alone does not explain EDEN’s success; rather, the EDN encoding provides richer, lower-dimensional inputs that enable this efficient network to focus its limited capacity on discriminative pattern recognition rather than constructing useful representations from sparse inputs.

To statistically validate these improvements, we conducted paired t-tests comparing EDEN against the OneHot + HFBranch baseline for each task, pooling results across all datasets within each task. Table 7 summarizes the statistical significance of EDEN’s improvements.

**Table 7:** Statistical significance of EDEN’s improvements over the OneHot + HFBranch baseline

Task	#Datasets	#Pairs	t-statistic	*p*-value
H-CPD	3	15	2.72	0.016
H-PD	3	15	2.58	0.005
H-TFP	5	25	4.73	0.0002
M-TFP	5	25	4.52	< 0.0001

For each task, paired t-tests were conducted on pooled MCC values from all datasets within that task (with 5 independent runs per dataset). The table shows the number of datasets, total paired observations (datasets × 5 runs), t-statistic, and corresponding p-value. All improvements are statistically significant at *p* < 0.05

The statistical tests confirm that EDEN’s performance gains, though modest in absolute MCC values (2.5–6.0%), are statistically significant across all tasks, with p-values ranging from 0.016 to < 0.0001. This robustness addresses potential concerns about the meaningfulness of small improvements in classification metrics.

The performance profile of EDEN is particularly notable for its task generality. While several models exhibit substantial task-specific specialization—for instance, DNABERT-2 excels in transcription factor prediction (Rank 1 in H-TFP and M-TFP) but shows weaker performance in promoter detection—EDEN maintains robust and balanced performance across the entire spectrum of genomic classification challenges. This generalizability makes EDEN particularly valuable for practical applications where consistent performance across multiple detection and classification tasks is essential, without requiring task-specific architecture modifications or extensive hyperparameter tuning.

### Discussion

The experimental results demonstrate that EDEN substantially improves DNA sequence classification across tasks and species. The core contribution is the multiscale EDN representation, which transforms symbolic sequences into numerical, position-aware density profiles, capturing both fine-grained motifs and broader compositional patterns that are often lost in traditional encodings. Paired t-tests confirm that EDEN’s gains over the OneHot + HFBranch baseline, though modest in absolute MCC values (2.5–6.0%), are statistically significant across all tasks (p-values from 0.016 to < 0.0001, Table 7).

To isolate the contribution of the encoding from model architecture, we introduced two baselines: OneHot + HFBranch (same CNN architecture) and K‑merFreq + XGBoost (one of the most common ML model for K‑mer Frequency classification). EDEN significantly outperformed both, demonstrating that improvements stem from the EDN encoding rather than the CNN backbone. While transformers like DNABERT‑2 excel in certain tasks, EDEN achieves competitive performance with orders of magnitude fewer parameters, highlighting the representational efficiency of EDN.

EDN differs fundamentally from convolutional smoothing: it is a deterministic, analytically defined representation computed before learning, encoding explicit multiscale nucleotide density. Convolutional filters, by contrast, are learned parameters that may not correspond to biologically meaningful patterns.

Biologically, EDN’s interpretability stems from its mapping to genomic principles (Sect. 3.4). Fine scales (~ 1‑mer) capture single-nucleotide preferences; intermediate scales (~ 3‑5‑mer) model core motifs; broader scales (~ 7+‑mer) represent regulatory domains. This structure provides a natural framework for linking predictions to sequence features. While comprehensive saliency and motif analyses remain for future work, EDN’s design inherently supports such interpretability investigations.

It is worth noting that EDEN addresses sequence-level classification, which differs from base-resolution transcription factor binding site prediction methods such as BPNet, NLDNN, FCNsignal, and FCNA. While those models predict exact binding positions and intensities at nucleotide resolution, EDEN classifies entire sequences for regulatory potential. These approaches are complementary: EDEN’s multiscale density representation could potentially inform base-resolution models by providing contextual sequence composition features.

In summary, EDEN demonstrates that integrating deterministic, interpretable representations with modern neural architectures yields both computational and biological advantages. EDN provides a multiscale view of sequence composition that complements learning‑based approaches, offering a foundation for future genomic analysis.

### Limitations

While EDEN demonstrates strong performance and interpretability potential across multiple regulatory sequence prediction tasks, several limitations warrant discussion:

- *Fixed yet biologically motivated bandwidths* EDN uses a set of predefined bandwidths (1-, 3-, 5-, 7-mer) selected via a principled decay criterion. These scales correspond to known biological feature sizes and have proven effective across all benchmarked tasks. While adaptive bandwidth learning could offer added flexibility for tasks with less clearly defined scales, the current approach provides a robust and interpretable baseline that does not require task-specific tuning.
- *Interpretability awaiting empirical validation* EDN is designed to be interpretable and is motivated by clear biological principles (Sect. 3.4). However, comprehensive empirical validation through saliency‑based methods (e.g., Integrated Gradients) or filter‑level motif extraction remains for future work.
- *Computational overhead in preprocessing* Computing multiscale EDN features introduces preprocessing overhead compared to simpler encodings like one‑hot vectors. Although the cost scales linearly with sequence length and can be parallelized, whole‑genome applications may require further optimization.
- *Sequence‑level focus* EDEN is currently designed for sequence‑level classification rather than base‑resolution prediction. As a result, it does not yet leverage the full positional precision exploited by architectures tailored for base‑resolution prediction, such as BPNet or other profile‑prediction models. Integrating EDN with such architectures—for instance, BPNet or FCNsignal —could further enhance fine‑grained interpretability and regulatory‑mechanism analysis.
- *Scope of biological validation* EDEN has been evaluated on sixteen datasets derived from human and mouse across four genomic tasks. Broader validation—on tasks such as enhancer prediction, splicing regulation, RNA‑binding site detection, or chromatin‑accessibility prediction—would further establish its generality.

## Conclusion

EDEN provides an efficient, accurate, and theoretically interpretable framework for DNA sequence classification. By integrating a novel encoding strategy with a streamlined hybrid architecture, it advances genomic classification, achieving performance competitive with state-of-the-art models while using orders of magnitude fewer parameters. The biologically grounded EDN encoding offers a structured, multiscale view of sequence composition, establishing a clear pathway for mechanistic interpretation beyond prediction accuracy. In summary, EDEN represents a step toward more parameter-efficient and inherently explainable deep learning for genomics.

## Future work

Building on EDEN’s performance, several research directions can extend its utility in computational biology:

- *Exploration of adaptive scale selection* Although the current fixed-bandwidth heuristic is effective and interpretable, future work could investigate adaptive or learnable bandwidth mechanisms for tasks where the relevant biological scales are not known a priori or vary across genomic contexts.
- *Architectural evolution with attention and transformers* Integrate attention mechanisms or transformer-based components to better capture long-range dependencies and global context, enabling dynamic weighting of multiscale features and sequence positions.
- *Advancing to quantitative genomic prediction* Apply EDEN to quantitative regression tasks—such as predicting chromatin-accessibility intensity (ATAC-seq/DNase-seq) or transcriptional output (CAGE/PRO-cap)—to model not only the presence of regulatory elements but also their functional strength.
- *Generalization to multi-modal biological sequences* Extend the density-based EDN principle to RNA sequences (e.g., secondary-structure prediction), protein sequences (capturing physicochemical properties), and epigenetic tracks (e.g., methylation density), positioning EDEN as a unified multi-modal representation tool.
- *Empirical interpretability validation* Conduct formal saliency-based analyses—such as Integrated Gradients and filter-level motif extraction—to empirically validate which sequence patterns are captured by each branch of EDEN and to link EDN’s multiscale features to known biological motifs and regulatory syntax.
- *Base‑resolution modeling for regulatory syntax* Combining EDN with base‑resolution architectures (e.g., BPNet‑style models or FCNsignal) would enable multiscale density features to inform precise, nucleotide‑level predictions, opening new avenues for deciphering regulatory grammar and the mechanistic impact of individual variants.

Collectively, these directions underscore EDEN’s potential to evolve from an efficient DNA classifier into a foundational, interpretable, and multi-modal framework for biological sequence analysis.

## Data Availability

All datasets used in this study are publicly available as part of the Genome Understanding Evaluation (GUE) benchmark. The benchmark datasets can be accessed at: https://huggingface.co/datasets/leannmlindsey/GUE. The full implementation of EDEN, including source code, pretrained models, and datasets are publicly available at https://github.com/zabihis/EDEN.

## Abbreviations

EDN: Expected Density of Nucleotide
EDEN: Expected Density of Nucleotide Encoding Framework
KDE: Kernel Density Estimation
CNN: Convolutional Neural Network
MCC: Matthews Correlation Coefficient
TF: Transcription Factor
TFP: Transcription Factor Prediction
HFBranch: High-Resolution Branch
GUE: Genome Understanding Evaluation
AE: Autoencoder
LLM: Large Language Model
ML: Machine Learning

## Declarations

### Image integrity

This study does not include gels, blots, or microscopy images, and therefore no raw image files are required.
